# Macronutrient composition of nickel-treated wheat under different sulfur concentrations in the nutrient solution

**DOI:** 10.1007/s11356-015-5823-6

**Published:** 2015-11-23

**Authors:** Renata Matraszek, Barbara Hawrylak-Nowak, Stanisław Chwil, Mirosława Chwil

**Affiliations:** Department of Plant Physiology, University of Life Sciences in Lublin, Akademicka 15, 20-950 Lublin, Poland; Department of Agricultural and Environmental Chemistry, University of Life Sciences in Lublin, Akademicka 15, 20-950 Lublin, Poland; Department of Botany, University of Life Sciences in Lublin, Akademicka 15, 20-950 Lublin, Poland

**Keywords:** Macronutrient content, Nickel stress, Sulfur nutrition, Wheat (*Triticum aestivum* L.)

## Abstract

The effect of different sulfate(VI) sulfur (2, 6, and 9 mM S) levels and nickel(II) chloride (0, 0.0004, 0.04 and 0.08 mM Ni) in the nutrient solution on productivity and macronutrient (N, P, K, Ca, Mg, S) status and accumulation in spring wheat (*Triticum aestivum* L.) Zebra cv. was studied. Ni treatment reduced the biomass and disturbed the balance and accumulation of macronutrients in wheat. Intensive S nutrition, especially with 6 mM S, at least partially increased the biomass, improved ionic equilibrium, and enhanced nutrient accumulation in Ni-exposed plants in spite of increased Ni accumulation. Admittedly, the dose 9 mM S reduced Ni accumulation in shoots but increased accumulation thereof in roots. Compared to 6 mM, the dose 9 mM was less effective in improving the mineral status of Ni-treated wheat.

## Introduction

Nickel (Ni) is recognized as a heavy metal micronutrient required for proper plant growth and development (Chen et al. 2009; da Silva et al. 2012a, b; Mazzafera et al. 2013). The Ni requirement of plants, which is below 0.5 mg kg^−1^ dry weight (DW), is the lowest of all essential elements (Liu et al. 2011; López and Magnitski 2011). Ni is a functional constituent of some enzymes inter alia urease (Sigel et al. 2007; Ragsdale 2009). The metabolism of this element is crucial for maintaining a proper cellular redox state and various other biochemical, physiological, and growth responses. Besides involvement in nitrogen (N) metabolism and iron absorption, Ni is required for viable seed production and germination (Brown 2007; Ahmad and Ashraf 2011; Poonkothai and Vijayavathi 2012).

During the last few decades, a significant increase in environmental contamination with Ni has been observed; hence, a phytotoxic effect of this element, rather than deficiency, is much more commonly found. The increasing Ni pollution of the environment is mainly caused by various anthropogenic activities: fossil fuel combustion, metal (especially Ni) ore mining, smelting and refining, metallurgical and electroplating industry, cement and steel manufacturing, municipal refuse incineration, electrical and electronic industry, chemical and food industry, agricultural use of sewage sludge, application of organic and mineral fertilizers, and many others (Cempel and Nikel 2006; Iyaka 2011; Wuana and Okieimen 2011; Yusuf et al. 2011). Ni pollution has become a serious concern. It is estimated that concentrations of this metal in polluted soils, surface waters, and air have reached up to 26,000 mg kg^−1^; 0.2 mg L^−1^; and 2000 ng m^−3^, respectively, which is 20–30 times higher than those found in unpolluted areas (Kabata-Pendias and Mukherjee 2007). The maximum permissible Ni concentration in agricultural soil according to the standards set by United Nations Economic Commissions for Europe (UNECE) is 100 mg kg^−1^ and in ground water, 20 μg L^−1^ (Gaillardet et al. 2005; Nazir et al. 2015). Soil clean-up target levels (SCTLs) have been determined to be 100 and 28,000 μg L^−1^ for residential considerations and commercial sites, respectively (Lander 2003). Ni moves through the environment very easily and is readily taken up by plants. Excessive concentrations of this element are phytotoxic and lead to severe growth inhibition and limited biomass production. The toxic Ni content in plants varies in relation to the degree of sensitivity or tolerance to the metal. It is assumed that a critical toxicity Ni level in sensitive, moderately tolerant, and tolerant species is 10, 50, and 100 mg kg^−1^ dry mass (DM), respectively (Kozlow 2005; Yusuf et al. 2011; Hussain et al. 2013). In hyperaccumulators (genus *Alyssum* and *Thlaspi*), the toxic Ni content exceeds 1000 mg kg^−1^ DM (Küpper et al. 2001; Pollard et al. 2002; Yusuf et al. 2011; Leitenmaier and Küpper 2013). The toxicity of Ni has become a worldwide problem threatening sustainable agriculture (Aydinalp and Marinova 2003; Kucharski et al. 2009). Cereals (especially oat) are recognized as very sensitive to Ni, whereas legumes and members of the mustard family can tolerate and accumulate high amounts of this element. There is little information about Ni toxicity mechanisms in plants, compared to other toxic trace metals like lead (Pb), cadmium (Cd), copper (Cu), and chromium (Cr). This is due to the dual character and complex electronic chemistry of this metal, which makes it difficult to study its biological role and toxicity (Kabata-Pendias and Mukherjee 2007; Yusuf et al. 2011). Toxic effects of Ni are observed at multiple levels. One of them is disrupting the nutritional status of plants. Ni interferes with uptake, transport, and distribution of elements of macro- and micronutrients. Literature data show a contradictory effect of Ni on plant mineral nutrition. The contents of mineral nutrients in organs of Ni-treated plants may drop, rise, or remain unchanged (Sreekanth et al. 2013). However, although there are many reports concerning the phytotoxic effects and tolerance to Ni, our knowledge in this area is still incomplete, and the detailed mechanisms involved are poorly understood.

Sulfur (S) is a macronutrient receiving special attention in soil science and plant nutrition. This element is involved not only in proper growth and development, but is also associated with biotic and abiotic stress tolerance in higher plants (Starast et al. 2007). The requirement of plants for this element ranges from about 0.1 to 1.0 % (on a dry weight basis). Wheat, the biological object of our investigations, is a species characterized by low requirements for sulfur (Droux 2004; Zagorchev et al. 2013). Together with nitrogen, sulfur is assimilated by plants in redox processes and forms part of carbohydrate compounds. Nowadays, progressive process of reducing emissions of S to the natural environment is observed. N and P fertilizers without S are much more frequently used than those containing S. Sulfate ions easily leach deeper into the soil profile and they are relatively immobile in the soil-plant system. All of the above-mentioned reasons limit S availability to plants and cause its deficiency and reduction of crop yield and quality (Mašauskiene and Mašauskas 2012). According to biochemical function, S, together with N, is classified as a nutrient forming the organic compounds of plants. S serves many functions in plants. This element is used in the formation of amino acids (cysteine (Cys), cystine (Cys)_2_, and methionine (Met)); peptides; proteins; lipoic acid (LA); essential oils (adenosine 5ʹ-phosphosulfate (AMPS)); and glucosinolate (3ʹ-phosphoadenosine-5ʹ-phosphosulfate (PAPS)) and is known as a universal S donor for sulfotransferases, or it is a structural component of these compounds. S is active in the conversion of inorganic N into protein. This element catalyzes chlorophyll formation. It promotes nodulation in legumes, helps develop and activate various enzymes, coenzymes, and vitamins (biotin, thiamin, coenzyme A (CoA)), and is their component. Ligands containing sulfhydryl (SH) groups, i.e., glutathione (GSH) or phytochelatins (PCs), form high-strength, durable complexes with heavy metals. It is claimed that especially the former compound plays an important role in Ni resistance (Bhatia et al. 2005; Hawkesford and De Kok 2006; Kopriva 2007; Khan et al. 2008; Gill and Tuteja 2011; Mazid et al. 2011a, b; Hossain et al. 2012; Viehweger 2014).

Given the fact that Ni, like most heavy metals, interferes with the uptake and transport of many essential nutrients including sulfur as well as taking into account the role of S in building resistance to stress caused by the presence of heavy metals, these studies were undertaken to assess the effect of intensive S–SO_4_^2−^ nutrition on the mineral composition of Ni-treated wheat (*Triticum aestivum* L.). This species is a very important agricultural crop showing a low demand for S. The results presented in this paper are only part of a research project concerning the role of intensive S nutrition in mechanisms of tolerance to Ni. We hope that our investigations will help to understand and develop strategies for alleviation of Ni phytotoxicity with additional sulfur fertilization.

## Materials and methods

### Plant material characteristics and growth conditions

The experiment was carried out in 2011–2014 in the Plant Physiology Department, University of Life Sciences in Lublin, Poland. The biological object of the study was spring wheat (*Triticum aestivum* L.), family Poaceae. For the experiment, an elite highly fertile Swedish bread cultivar Zebra (Firm SVALOV WEIBULL) with superior baking quality and high tolerance to pathogens, except for leaf rust (*Puccinia recondita* Rob. ex Desm. f. sp. *tritici* (Eriks.) Jonson), was chosen. The examined cultivar Zebra was included into the Polish National List in COBORU in 2000 (Polish National List of Agricultural Plant Varieties 2012). Plants of this cultivar are medium in height with a very good lodging resistance and quite an early deadline of heading and ripening. The grain of this variety is medium-sized with average alignment. It contains high levels of best-quality protein and gluten. The offal content is very low. “Zebra” is a variety with moderate soil requirements and much better resistance to drought than other cultivars. A factor significantly limiting yielding of this cultivar is low pH.

The experiment was carried out with the method of water cultures. One-week-old seedlings were transferred to 1 dm^3^ glass jars (two plants each) with Hoagland’s II solution. The differentiating factors of the experiment were the sulfur level = 2.00 (control, basic, or standard dose), 6.00, and 9.00 mM and nickel concentration (NiCl_2_) = 0, 0.0004, 0.04, and 0.08 mM. The standard sulfur dose (2 mM) was supplied as MgSO_4_, while in the treatment with high S doses, i.e., 6 and 9 mM S, a standard sulfur dose in the MgSO_4_ form was additionally supplemented with appropriate amounts of Na_2_SO_4_. In all experimental treatments, the level of sodium (Na) and chlorine (Cl) was equalized by adding appropriate amounts of 1 % NaCl and HCl to the nutrient solution; the pH of the nutritional environment was set at 5.8–6.0. Plant vegetation was conducted in a phytotron under controlled conditions: 25:20 °C day/night temperature, 14:10 h photoperiod, photosynthetic photon flux density (PPFD) 400 ± 10 μmol m^−2^ s^−1^, and relative air humidity between 60 and 70 %. After 2 weeks of growth under conditions of different Ni contamination and S nutrition, plants were harvested and dry mass (DM) as well as the macronutrient composition of roots and shoots (total nitrogen (N), phosphorus (P), potassium (K), calcium (Ca), magnesium (Mg), total sulfur (S) content) was assessed.

### Macronutrient content and accumulation

The dry plant material (roots and shoots separately) was subjected to chemical analyses according to well-known and commonly used procedures: the classic Kjeldahl procedure for the determination of total N, the molybdenum vanadate technique for total P, the flame-photometric method (atomic absorption spectrometry (AAS)) for K and Ca, the colorimetric method with the use of titan yellow for Mg, and the nephelometric Butters-Chenery method for estimating the total S content. The determination of N and P was performed after wet mineralization in sulfuric acid. The results concerning the macronutrient content were used to calculate the accumulation of macronutrients and their ratios (K/(Ca + Mg), Ca/Mg, Ca/P, N/S − ratio). The accumulation of the nutrient content was calculated by multiplying dry weight (DW) of plants by the concentration of each nutrient in the biomass (Bessa et al. 2013).

### Nickel determination

Analyses of the Ni content in roots and shoots was carried out using the classic atomic AAS method, following prior dry mineralization of 5.000 g plant samples at 500 °C, dissolved in 20 % HNO_3_. Ni analyses were performed by an accredited laboratory of the Regional Chemical-Agricultural Station in Lublin. In this work, data concerning the Ni accumulation have been presented. The total amount of Ni accumulated in under- and aboveground parts were calculated based on the concentration of this metal in the plant parts and dry matter yields (Pereira et al. 2011).

### Statistical analysis

The experiment covered 12 treatments, 20 repetitions in each treatment, and 3 repetitions over time. The results of chemical analyses obtained were processed statistically by the two-way analysis of variance (ANOVA) using the STATISTICA 9 software (StatSoft, Inc. 2009). The experimental factors were the level of S nutrition of plants and the Ni content in the nutritional environment. Mean values were compared by the Tukey’s post hoc test and the differences were considered significant at *P* ≤ 0.05. Comparison of values in the same treatment as well as mean values among each treatment obtained from each of the three independent replicates of the experiment over the time did not show statistically proven differences. Therefore, the data presented in the tables and in the figures represent mean values obtained from nine measurements (three measurements made per each independent repetition of the experiment over the time).

## Results

### Dry matter

**Fig. 1:**
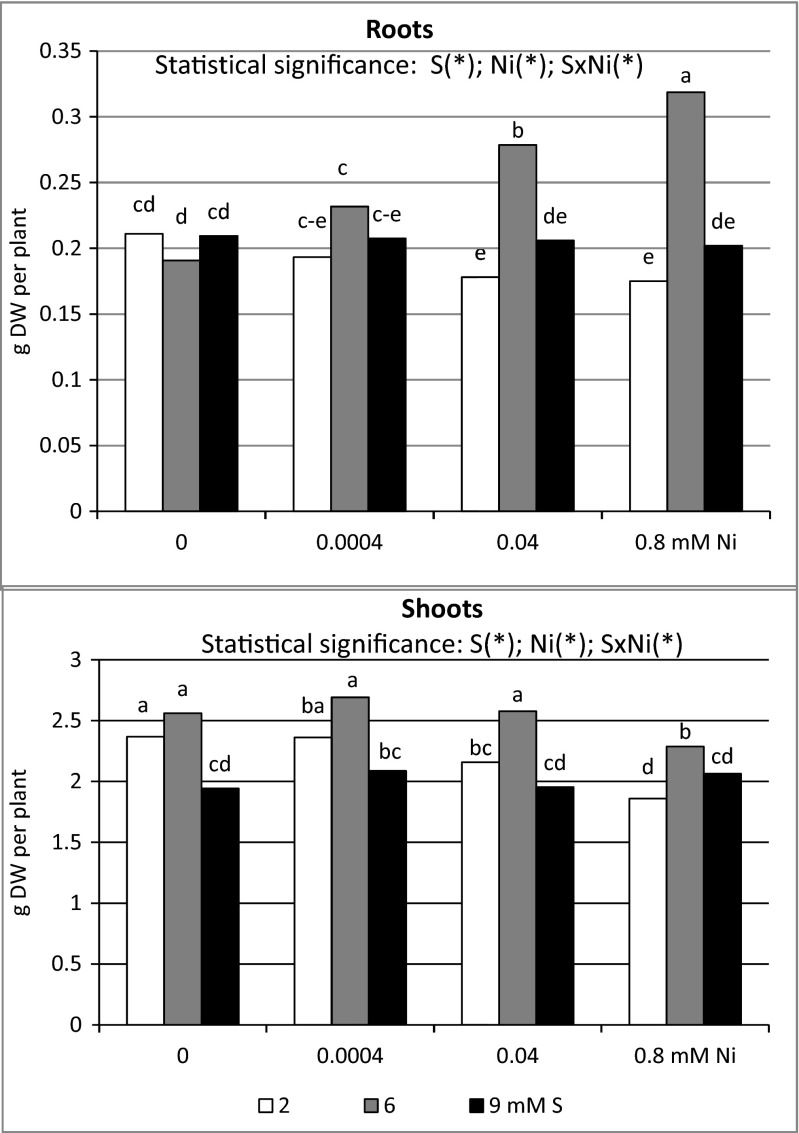
Dry yield matter of spring wheat cv. Zebra grown under different sulfur (S) and/or nickel (Ni) concentrations in the nutrient solution. Results are mean of nine replications. Means marked by the *same letter* are not different at *P* ≤ 0.05 based on the Tukey’s honestly significance test. Significant effects for the main factors and for the interaction between them are indicated with *asterisks*

Irrespective of the S level in the nutrient solution, the presence of increasing Ni concentrations (0.0004–0.08 mM) significantly elevated wheat root biomass (Fig. 1). At the same time, the lower Ni doses (0.0004 and 0.04 mM) did not change markedly, but the highest dose of this element used (0.08 mM) decreased shoot biomass. However, Ni presence in the nutrient solution at the standard S dose (2 mM) resulted in a significant drop in the root and shoot biomass (Fig. 1).

Under the intensive S nutrition with the dose 6 mM, there was a significant increase found in the root biomass of the Ni-stressed wheat accompanied by the lack of statistically proven changes of shoot biomass at the lower and medium Ni concentration and significant decrease of under the highest Ni concentration used in the experiment. Simultaneously, the dose 9 mM S did not change the root and shoot productivity defined as the amount of dry matter produced by plant during the vegetation (Fig. 1).

There was a significant effect of S and Ni interaction on the productivity of under- and aboveground wheat parts (Fig. 1). It was shown that high S supplementation of Ni-exposed plants, as compared to standard dose 2 mM S in general significantly raised (6 mM) or did not substantially change (9 mM S) the dry weigh of roots and shoots.

### Macronutrient content and accumulation

The results indicated that the Ni presence in the nutrient solution (0.0004, 0.04, and 0.08 mM), irrespective of the S level, substantially reduced the P, K, Ca, Mg, and S content in wheat shoots, except for the insignificant changes in the Mg content at the low Ni contamination level. Simultaneously, the N content in the aboveground parts of the Ni-stressed wheat increased at the lowest, remained quite stable at the medium, and dropped at the highest Ni contamination (Table 1). In turn, in the roots, a decrease in the K and Ca content was found, together with insignificant changes in the contents of N, P, Mg, and S. An exception was the substantial drop in the N content at 0.08 mM Ni and the decrease in the Mg content at 0.04 mM Ni as well as the lack of statistically proven changes in the K content under the 0.08 mM Ni and Ca content under 0.04 mM Ni (Table 1). Moreover, it was shown that the increasing Ni level in the nutrient solution, irrespective of the S level, significantly decreased P, K, and S bioaccumulation, but did not markedly affect the bioaccumulation of Mg. Only changes in S accumulation in wheat treated with 0.08 mM Ni were insignificant (Table 2). Simultaneously, Ni doses 0.0004 and 0.04 mM did not significantly affect the accumulation of N and Ca, but the Ni concentration 0.08 mM substantially decreased the accumulation of both these macronutrients in the wheat biomass (Table 2).

**Table 1:** The content of macronutrients (g kg^−1^ DM) in the biomass of spring wheat cv. Zebra grown under different sulfur (S) and/or nickel (Ni) concentrations in the nutrient solution

Concentration (mM)		N		P		K		Ca		Mg		S	
Sulfur (S)	Nickel (Ni)	Roots	Shoots	Roots	Shoots	Roots	Shoots	Roots	Shoots	Roots	Shoots	Roots	Shoots
2		14.51d	25.19a	3.67a–c	3.72ab	26.38bc	44.29ab	18.99a–c	4.03a	39.74cd	3.59a	14.63ab	5.72b–d
6	0.00	16.24a–c	22.04e	3.59a–c	3.49ab	27.79ab	44.39ab	17.09c	3.17b–d	41.56a–c	3.25a–c	12.52b–d	5.79b–d
9		14.97cd	21.18c–e	3.88ab	3.65ab	28.15a	45.56b	20.08a	2.89c–e	41. 12cb	2.73d–f	13.75a–d	6.91ab
2		15.12bd	24.58ab	3.99a	2.24cd	28.04b	39.48c–e	17.30c	3.24cd	38.17de	3.04b–e	11.63d	5.03c–e
6	0.0004	15.87bd	25.49a	3.89ab	3.84a	24.56d–f	41.27bc	17.89bc	3.71ab	40.36b–d	3.32ab	15.25a	4.88de
9		15.02bd	23.85a–c	4.37a	3.48ab	24.35d–c	40.67cd	17.98bc	3.52a–c	39.75cd	3.28a–c	15.38a	6.28a–c
2		13.02e	20.73de	4.31a	2.18d	25.94cd	35.69fg	19.83ab	2.52e	42.34ab	2.79c–f	11.06cd	4.34e
6	0.04	17.45a	23.17a–d	4.41a	3.17a–c	23.71ef	40.78cd	18.85bc	3.26b–d	38.56d	3.14a–d	14.13a–c	5.13c–e
9		16.39ab	21.17c–e	3.07bc	2.41cd	22.97f	37.28ef	19.02a–c	2.79de	35.18f	2.85b–f	15.00a	7.34a
2		10.06f	17.42f	4.06a	1.87d	31.78a	30.22h	11.23d	2.47e	41.95a–c	2.21g	14.11a–c	4.83de
6	0.08	16.19a–c	21.90b–e	2.99c	2.79b–d	25.43c–e	37.49d–f	19.86a–b	2.96c–e	43.75a	2.56e–g	15.56a	5.69b–d
9		15.35b–d	19.89ef	3.58a–c	2.34cd	24.45d–f	32.68gh	18.26a–c	2.52e	36.02ef	2.45fg	12.34b–d	7.28a
Main effects													
S concentration													
2 mM		13.18c	21.98b	4.01	2.50b	28.04a	37.42c	16.85b	3.07	40.55a	2.91	12,85b	4.98c
6 mM		16.44a	23.15a	3.72	3.32a	25.37b	40.73a	18.42a	3.28	41.06a	3.07	14.36a	5.37b
9 mM		15.43b	21.52b	3.73	2.97ab	24.98b	39.05b	18.84a	2.93	38,02b	2.83	14,12a	6.95a
Ni concentration													
0 mM		15.24a	22.80b	3.71	3.62a	27.44a	44.75a	18.72a	3.36a	40.80a	3.19a	13.63	6.14a
0.0004 mM		15.34a	24.64a	4.08	3.19b	25.65b	40.47b	17.74b	3.49a	39.43ab	3.21a	14.09	5.40b
0.04 mM		15.62a	21.69b	3.93	2.59c	24.21c	37.92c	19.23a	2.86b	38.69b	2.93a	13.40	5.60b
0.08 mM		13.87b	19.74c	3.54	2.33c	27.22a	33.46d	16.45c	2.65c	40.57a	2.41b	14.00	5.93c
Statistical significance													
S concentration		*	*	NS	*	*	*	*	NS	*	NS	*	*
Ni concentration		*	*	NS	*	*	*	*	*	*	*	NS	*
S × Ni concentration		*	*	*	*	*	*	*	*	*	*	*	*

Different letters within the same column indicate significant differences between means of nine replications according to the Tukey’s multiple range test (*P* ≤ 0.05)

*NS* not significant

**Table 2:** Macronutrients accumulation (mg per plant) in the biomass of spring wheat cv. Zebra grown under different sulfur (S) and/or nickel (Ni) concentrations in the nutrient solution

Concentration (mM)							
Sulfur (S)	Nickel (Ni)	N	P	K	Ca	Mg	S
2		62.80b	9.58ab	110.44ab	13.55ab	16.88b–d	16.63a
6	0.00	59.54bc	9.62ab	116.42a	11.37bc	16.25c–e	17.21a
9		44.26d	7.90b	94.37c	9.82c	13.91d–g	16.30ab
2		60.98b	6.06de	98.68bc	11.00bc	14.56d–f	14.13b
6	0.0004	73.39a	11.51a	118.48a	15.38a	21.11a	17.73a
9		42.15d	6.60d	71.62ef	9.49c	13.62e–g	13.47c
2		47.05d	5.47de	81.63de	8.96c	13.55e–g	11.33c
6	0.04	64.57b	9.40a–c	111.69a	13.65ab	18.83a–c	17.15a
9		44.73d	5.34de	77.55e	9.36c	12.81fg	17.43a
2		34.15e	4.19e	61.76f	6.56d	11.45g	11.45c
6	0.08	55.26c	7.33cd	93.86cd	13.10a	19.79ab	17.97a
9		44.14d	5.44de	72.37ef	8.89cd	12.33fg	17.51a
Main effects							
S concentration							
2 mM		51.22b	6.32b	88.13b	10.02b	14.11b	13.39c
6 mM		63.19a	9.46a	110.12a	13.37a	18.99a	17.52a
9 mM		43.83c	6.35b	78.99c	9.39b	13.16b	16.18b
Ni concentration							
0 mM		55.51ab	9.04a	107.09a	11.58a	15.68	16.72a
0.0004 mM		58.84a	8.06a	96.26b	11.96a	16.43	15.11b
0.04 mM		52.12b	6.73b	90.29b	10.66ab	15.06	15.30b
0.08 mM		44.52d	5.69b	76.00c	9.51b	14.52	15.64ab
Statistical significance							
S concentration		*	*	*	*	*	*
Ni concentration		*	*	*	*	NS	*
S × Ni concentration		*	*	*	*	*	*

Different letters within the same column indicate significant differences between means of nine replications according to the Tukey’s multiple range test (*P* ≤ 0.05)

*NS* not significant

As a result of intensive S nutrition (6 and 9 mM S) of the Ni-treated spring wheat (0.0004–0.08 mM), an increase in the root N content together with a substantially unchanged root content of P was observed. Simultaneously, the shoot content of both these macronutrients increased at 6 mM and remained quite stable at 9 mM S (Table 1). It was also shown that the high S level in the nutrient medium of Ni-exposed wheat markedly reduced the K content in the roots and raised it in the shoots, while at the same time, the root Ca content increased but the shoot content of this macronutrient did not change substantially. Moreover, the results obtained indicate that, depending on the S dose, the root Mg content of the Ni-contaminated plants did not changed substantially (6 mM) and dropped (9 mM). Simultaneously, no significant change in the shoot Mg content was recorded. Furthermore, it was found that the shoot and root S content in the Ni-stressed wheat intensively supplied with S raised significantly (Table 1).

The macronutrient accumulation in the wheat biomass under Ni treatments at different S levels depended on the S dose rather than on the Ni concentration in the nutrient solution (Table 2). Intensive S nutrition with the dose 6 mM under conditions of Ni presence resulted in a significant increase in bioaccumulation of all macronutrients, while the S dose 9 mM S substantially increased S and decreased N and K accumulation but P, Ca, and Mg accumulation was not significantly changed (Table 2).

There was a significant effect of S and Ni interaction on the root and shoot content of macronutrients as well as on accumulation thereof in wheat (Tables 1 and 2). It worth to stress the increase in N and S as well as decrease in K and Mg contents recorded in roots of Ni-exposed plants intensive fertilized with S. Simultaneously, root P content did not change significantly at 0.0004, raised at 0.04 and dropped at the concentration 0.08 mM Ni. In turn, in shoots of Ni-exposed wheat intensive supplied with S, the significant increase in P and Ca content accompanied by the slight changes in N and Mg content were recorded. The exceptions were insignificant changes in P and Ca content recorded at the highest Ni concentrations used in the experiment. It was shown that high S supplementation of Ni-exposed plants, in general, significantly raised at 6 mM and did not substantially change at the S level 9 mM the K content in shoots, while shoot S content remained unchanged and elevated, respectively. Furthermore, it was shown that the intensive S nutrition of Ni-exposed plants, in general, significantly raised (6 mM) or did not change markedly (9 mM S) the bioaccumulation of N, P, K, Ca, and Mg excluding increase in N bioaccumulation shown for the treatment 9 mM S/0.08 mM Ni. All these changes were accompanied by the rise in S bioaccumulation.

### Macronutrient ratios

Irrespective of the S level in the nutrient solution, the increasing Ni contamination did not affect the N/S ratio in wheat roots and shoots (Table 3). Simultaneously, the shoot K(Ca + Mg) ratio was substantially lower, while the value of this ratio in roots at the 0.0004 and 0.08 mM Ni ratio was quite stable, but significantly dropped at 0.04 mM Ni. The increasing Ni level in the nutrient solution did not cause significant changes in the shoot Ca/Mg ratio, but the value of this ratio in roots was reduced at 0.0004 and 0.08 mM Ni and was elevated at 0.04 mM Ni. Under conditions of the low Ni concentration (0.0004 mM), intensive S fertilization resulted in a decrease in root Ca/P, but at the high Ni contamination (0.04 and 0.08 mM) the value of this ratio was unaffected. Simultaneously, in the Ni-stressed plants, irrespective of the S level in the nutrient solution, the shoot value of this ratio was substantially higher (Table 3).

**Table 3:** Ratios of macronutrients in the biomass of spring wheat cv. Zebra grown under different sulfur (S) and/or nickel (Ni) concentrations in the nutrient solution

Concentration (mM)		K/(Ca + Mg) mole		Ca/Mg mass		Ca/P mass		N/S mass	
Sulfur (S)	Nickel (Ni)								
		Roots	Shoots	Roots	Shoots	Roots	Shoots	Roots	Shoots
2		0.53d	2.28cd	0.48a–c	1.12ab	5.17b	1.08c	0.99e	4.40ab
6	0.00	0.60bc	2.67b	0.41de	0.98d	4.76cd	0.91ef	1.30a	3.81cd
9		0.54d	3.16a	0.49a–c	1.06bc	5.18b	0.79f	1.09cd	3.07e
2		0.61b	2.34b–e	0.38e	1.07bc	4.34ef	1.45a	1.30a	4.89a
6	0.0004	0.52d	2.13e	0.44cd	1.12ab	4.60de	0.97de	1.04de	3.25d-f
9		0.52d	2.19de	0.45b–d	1.07ab	4.11f	1.01c–e	0.98e	3.80cd
2		0.51d	2.56b–d	0.47b–d	0.90e	4.60de	1.16bc	1.18bc	4.78a
6	0.04	0.53d	2.39b–e	0.49a–c	1.04cd	4.27ef	1.03c–e	1.24ab	4.52a
9		0.51d	2.50b–e	0.54a	0.98d	6.20b	1.16bc	1.09cd	2.88ef
2		0.70a	2.43b–e	0.27f	1.12ab	2.77g	1.32ab	0.71f	3.61c-e
6	0.08	0.51d	2.60bc	0.45b–d	1.16a	6.64a	1.06c–e	1.04d	3.85bc
9		0.55cd	2.49be	0.51ab	1.03cd	5.10bc	1.08cd	1.24ab	2.73f
Main effects									
S concentration									
2 mM		0.59a	2.40	0.40b	1.05	4.22b	1.25a	1.05c	4.42a
6 mM		0.54b	2.45	0.45a	1.08	5.07a	0.99b	1.16a	3.86b
9 mM		0.53b	2.59	0.49a	1.04	5.15a	1.01b	1.10b	3.12c
0 mM		0.56ab	2.70a	0.46b	1.05	5.04a	0.93b	1.13	3.76
Ni concentration									
0.0004 mM		0.55bc	2.22c	0.42c	1.09	4.35b	1.14a	1.11	3.98
0.04 mM		0.52c	2.48b	0.50a	0.97	5.02a	1.12a	1.17	4.06
0.08 mM		0.59a	2.51b	0.41c	1.10	4.84a	1.15a	1.00	3.40
Statistical significance									
S concentration		*	NS	*	NS	*	*	*	*
Ni concentration		*	*	*	NS	*	*	NS	NS
S × Ni concentration		*	*	*	*	*	*	*	*

Different letters within the same column indicate significant differences between means of nine replications according to the Tukey’s multiple range test (*P* ≤ 0.05)

*NS* not significant

The results obtained indicate that the increased S level (6 and 9 mM) in the Ni-contaminated nutrient solution, irrespective of the Ni concentration, resulted in a decrease in the root K/(Ca + Mg) ratio, but in shoots, the values of this ratio were not significantly different (Table 3). Simultaneously, a significant increase in the root Ca/Mg, Ca/P, and N/S ratios was noticed, while in shoots, the value of the Ca/Mg ratio remained quite stable, but the Ca/P and N/S ratio was substantially lower (Table 3).

There was a significant effect of S and Ni interaction on the value of K/(Ca + Mg), Ca/Mg, Ca/P, and N/S in shoots and roots. The elevated S level in Ni containing nutrient solution significantly dropped the root K/(Ca + Mg) ratio and did not change the value of this ratio in shoots, except for insignificant changes in root shown for the medium Ni level used in the experiment (0.04 mM). At the same time, Ca/Mg ratio in roots of Ni-stressed wheat was increased under conditions of intensive S fertilization, while in shoots, was slightly changed at lowest (0.0004), increased at medium (0.04) and decreased at the highest (0.08 mM) used in the experiment Ni concentration. In turn, the root value of Ca/P ratio of objects intensive supplied with S was not markedly affected at 0.0004 mM and raised at the higher Ni concentrations in nutrient solution (0.04 and 0.08 mM), while in shoots, the value of this ration dropped at the lowest and at the highest but did not change at the medium Ni concentration used in the experiment. Furthermore it was shown that high S supplementation of Ni-exposed plants, in general, significantly raised, and the S dose 6 mM and did not substantially change at S level 9 mM the value of shoot N/S ratio, while the root value of this ratio substantially decreased at 0.0004, remained unchanged at 0.04 and increased at the concentration 0.08 mM Ni.

### Nickel accumulation

**Fig. 2:**
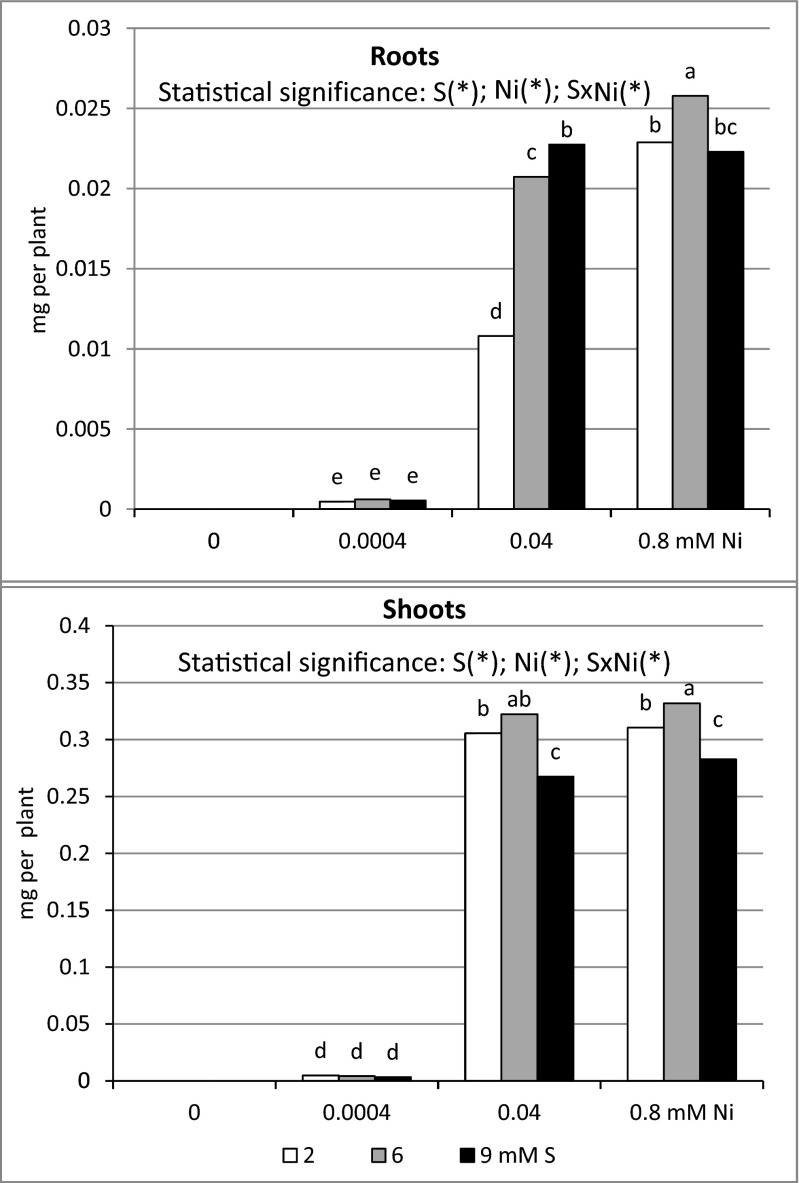
Nickel accumulation in the biomass of spring wheat cv. Zebra grown under different sulfur (S) and/or nickel (Ni) concentrations in the nutrient solution. Note: In control plants (Ni = 0 mM) in basic (S = 2) and high S (S = 6 or 9 mM) treated plants only trace amounts of nickel were identified and, hence, accumulations were zero. See Fig. 1 for further explanation.

The results obtained indicate that, irrespective of the S level in the nutrient solution, Ni accumulation in the spring wheat biomass rose together with the increasing concentration of this element in the nutrient medium and that the increase in Ni accumulation was much more pronounced in shoots than in roots (Fig. 2). Depending on the Ni level in the nutrient solution, wheat accumulated 7–10 (0.0004 mM), 16–28 (0.04 mM), and 13–14 times (0.08 mM Ni) larger amounts of Ni in shoots than in roots. Irrespective of the Ni level in the nutrient solution, the intensive S nutrition (6 and 9 mM) significantly increased Ni accumulation in roots. Simultaneously, shoot Ni accumulation remained quite stable at 6 mM, but dropped markedly at 9 mM S. There was a significant effect of S and Ni on the Ni accumulation in the under- and aboveground parts of wheat (Fig. 2). It is worthy to stress statistically proven increase in the Ni accumulation in the root biomass of intensive fertilized with S plants exposed to medium and the highest Ni concentrations used in the experiment, excluding the treatment 9 mM S/0.08 mM Ni where Ni accumulation remained quite stable. In turn, in the shoot biomass, the decrease in Ni accumulation shown in high Ni stressed (0.04 and 0.08 mM) objects supplemented with the S dose 6 mM should be mentioned.

## Discussion

The Ni concentration used in the present study ranged between 0.0004 and 0.08 mM. The lowest concentration of this micronutrient in the nutrient solution is regarded as the highest permissible level in ground water. Moreover, the dose 0.0004 mM Ni exceeds about eight times the background concentration of this element in ground water and 1.3 times the global average value. The level of Ni from water in industrial regions tends to be from 6 × 10^−4^ to 6 × 10^−2^ mM. For protection of aquatic organisms, Soil Quality Assessment Guideline (SQAG) established a value of threshold effect levels (TEL) at 3 × 10^−5^ mM and probable effect levels (PEL) at 75 × 110^−6^ mM (Lander 2003; Kabata-Pendias and Mukherjee 2007). The second experimental factor was various S–SO_4_ concentrations in the nutrient solution. The standard S–SO_4_ level used in the presented studies (2 mM S) is considered moderate. It is assumed that the S–SO_4_ concentration in the natural environment, i.e., unpolluted with heavy metals, ranges from 0.16 to 7, in arid regions from 3 to 16 mM, while the SO_4_^2−^ concentration in soil solutions with residues of sulfide ore mine oscillates between 13 and 110 mM (Ernst et al. 2008).

### Macronutrient content and accumulation

Disturbances in essential element balance and ionic homeostasis is considered a crucial mechanism of Ni toxicity in plants. The data concerning the Ni impact on plant mineral nutrition are inconclusive. Mineral nutrient content in particular organs of Ni-stressed plants may decrease, remain quite stable, or increase (Rubio et al. 1994; Sreekanth et al. 2013). The decrease in the content of all examined macronutrients found in the presented studies for the Ni-stressed wheat may be explained, inter alia, by low-energy status as a result of respiration disturbances, which decreases active uptake and nutrient transport. Due to the comparable ionic radii of Ni^2+^ and other cations of essential nutrients, they may compete for common biding sites. Ni may also turn off nutrients (especially Ca, Mg, Fe, Zn, Cu) from their physiological function. Replacement of the essential metal of metalloproteins, binding to catalytic residues of non-metalloenzymes, binding outside the catalytic site of an enzyme to inhibit allosterically, and causing oxidative stress are mechanisms of Ni toxicity often described in the literature (Chen et al. 2009; Gospodarek and Nadgórska-Socha 2010; da Silva et al. 2012a, b; Osu and Isaac 2014). Ni affects the composition and permeability of cell membrane by changing sterol and phospholipid levels as well as structural conformation and ATPase activity. Proton pump H-ATPase is involved in active uptake and transport of essential elements, which depends on the availability of metabolic energy utilized for cell membrane polarization. The indirect effect of Ni on enzyme activity arises from ion-induced imbalances due to competitive inhibition of absorption and transport of macro- and micronutrients, while the direct one involves strong Ni affinity for functional sulfhydryl groups (−SH) of enzymes and, in consequence, alteration of protein conformation thereby causing inactivation thereof and metabolic disorders (Janicka-Russak et al. 2008; Sanz et al. 2009; Sharma and Dhiman 2013; Sreekanth et al. 2013). Thus, Ni exposure alters sulfhydryl homeostasis. The primary route for Ni toxicity is depletion of GSH and bonding to the −SH groups of proteins. Ni tolerance and the detoxification process is associated with S metabolism, especially with high levels of OAS (O-acetyl-l-serine), Cys, and GSH in the biomass due to high activity of Ser acetyltransferase (SAR). Ni, which is a borderline metal, is able to bind with many types of chelating agents including S-donor ligands, i.e., containing highly reactive S functional groups which determine the durability of complexation (Bhatia et al. 2005; Seregin and Kozhevnikova 2006; Hossain et al. 2012; Viehweger 2014). Our findings revealed increased GSH content in the Ni-stressed wheat, elevated at 6 mM S, while the content of this tripeptide dropped at 9 mM S in the nutrient solution, which may suggest that the latter level was too high for this species (data in press). Also, application of the lower S dose used in the experiment (6 mM), irrespective of Ni contamination, gave much more beneficial effects on the nutrient composition in the wheat biomass compared to 9 mM. Admittedly, both S doses increased the N and S content in root and shoot biomass, and raised the Ca content in roots. In turn, the elevated P content was found only in the presence of 6 mM S. Moreover, the level 9 mM S significantly reduced the Mg content in roots. Simultaneously, both S doses decreased the K content in roots and increased it in shoots, whereas the changes in the content of this macronutrient in under- and aboveground parts were comparable. The data obtained confirmed that excess of S in the nutrient environment usually results in uptake of N in excessive amounts (de Kok et al. 2011). These processes can also induce ion imbalance in plants and disturbance of the buffer capacity of the cell sap (Fageira 2008). In all experimental treatments, the recorded leaf N, K, and S content exceeded the sufficient range of these elements, which is for N, K, and S are 2.5 to 3.5, 16 to 30, and 2 to 5 g kg^−1^ DW, respectively (Akhter 2012). Only the S content under conditions of Ni treatment (0.02 and 0.04 mM) at the basic S level (2 mM) lay within the upper limit of the optimal value. All the values of Mg, P, and Ca contents obtained in wheat aboveground parts oscillated within the optimal range, i.e., 1.3–4.0 g∙kg^−1^ DW for Mg and 2 to 5 g kg^−1^ DW for Ca and P (Akhter 2012).

The decrease in productivity and the reduced content of N, P, K, Ca, and S, shown in the presence of high Ni doses (0.04 and 0.8 mM) at the standard S level (2 mM) resulted in unfavorable changes in accumulation thereof. Except for Mg, the reduced accumulation of the analyzed macronutrients in the total biomass of the Ni-stressed wheat supplemented with the basic S dose resulted, to a greater extent, from the decrease in their content rather than the drop in productivity. In turn, Mg accumulation in the Ni-stressed wheat remained unchanged due to a comparably slight drop in the biomass and Mg content.

The positive effect of sulfur fertilization on macronutrient accumulation was shown at 6 mM S. Such a phenomenon was not evident at the highest additional S level, i.e., 9 mM. The increased accumulation of the macronutrients (N, P, K, Ca, and Mg) in the total biomass shown in the presence of Ni in the nutrient solution containing 6 mM S resulted from an increase in plant productivity rather than changes (mainly rise) in their content. N and K accumulation in the Ni-treated wheat fertilized with the highest experimental S dose (9 mM) was significantly reduced due to the greater drop in productivity than the rise in their content. At the same time, S accumulation rose mainly due to the marked increase in the S content. The unchanged P, Mg, and Ca accumulation in Ni-stressed plants at 9 mM S was caused by comparable changes in the content of this element and productivity.

### Macronutrient ratios

The quite stable value of the wheat root K/(Ca + Mg) ratio found in the presence of 0.0004 and 0.08 mM Ni resulted from the similar decreases in the K and Ca content as well as the slight changes in the Mg content. At the same time, under the Ni concentration 0.04 mM, the root value of this ratio was lowered as a result of the greater decrease in the K than Mg content together with the slightly changed Ca content. At the same time, the shoot K/(Ca + Mg) ratio in the Ni-stressed wheat plants was markedly lowered due to the greater decrease in the K than Ca content and the slightly changed Mg content. The changes in the root Ca/Mg ratio recorded in the Ni-stressed wheat, i.e., the drop at the 0.0004 and 0.08 mM Ni concentration as well as the rise at the 0.04 mM Ni dose resulted mainly from the changes in the Ca content, while the Mg content remained quite stable. At the same time, the slightly changed shoot Ca/Mg ratio was a consequence of the similar decrease in the Ca and Mg content. The quite stable root Ca/P ratio shown in the treatments contaminated with nickel, irrespective of the S level in the nutrient solution, was due to the comparable rise (at 0.0004 and 0.04 mM Ni) and drop (at 0.08 mM Ni) in the Ca and P content. In turn, the elevated value of this ratio in shoots resulted from the greater decrease in the P than Ca content. The quite stable value of the root N/S ratio found under the Ni treatment, irrespective of the S dose, was due to the unchanged N and S content. Only the drop in the N content recorded under the highest Ni contamination used in the experiment (0.08 mM) was significant. Also, the shoot N/S ratio remained unchanged. Depending on the Ni treatment, this was a consequence of the similar rise in S and the drop in the N content (at 0.0004 mM Ni) as well as the comparable drop in the content of both these macronutrients (at 0.04 and 0.08 mM Ni).

Our results revealed that in wheat fertilized with high S doses (6 or 9 mM), irrespective of the Ni treatment, the value of the root K/(Ca + Mg) ratio significantly dropped as a result of the decrease in the K content and the increased Ca content in spite of the slightly changed (at 6 mM S) and reduced (at 9 mM S) Mg content. The values of the shoot K/(Ca + Mg) obtained in the Ni-exposed wheat supplied with high S doses fall within the range of 2.13–2.60 and slightly exceeded the optimum range, which is 1.6–2.1:1, probably due to the fact that in order to maintain the ion balance, the cationic demand increases, and among the available K, Ca, and Mg ions in the nutrient medium, the K ion is much more rapidly taken up (Morgan and Connolly 2013). The shoot value in the Ni-stressed plants supplemented with additional S did not change markedly due to the slightly changed content of Mg at both S doses as well as the quite stable K and Ca content at 9 mM S and the similar increase in their content at 6 mM S. The significantly higher root Ca/Mg ratio in the plants supplemented with high levels of S, compared to those grown under conditions of the standard S level, was a consequence of the significant increase in the Ca content together with the unchanged (at 6 mM S) or decreased (at 9 mM S) Mg content. Simultaneously, the quite stable value of the shoot Ca/Mg ratio resulted from the slightly changed Ca and Mg content. In all experimental treatments, the value of the Ca/P ratio in aboveground wheat parts oscillated around the optimal value, which is 1 (Grzegorczyk and Gołebiewska 2004). Under the conditions of high S supplementation, irrespective of the Ni dose in the nutrient solution, the higher root Ca/P ratio recorded was related to the significant rise in Ca and the slight changes in the P content. In turn, the lower shoot Ca/P ratio in the treatments of the high S dose was a consequence of the increase in the P content together with the quite stable Ca content. It is generally recognized that the value of the N/S ratio indicates the status of S supply to plants. Such a statement is based on the fact that the vegetative organs of almost all crop plant species are characterized by quite a similar N/S value, which is 15:1 and the 10–15:1 range of this ratio is considered optimal (Blake-Kalff et al. 2000; Jez 2008; Jamal et al. 2010). The value of the N/S ratio recorded in the presented studies for wheat shoots oscillated in the range from 2.73 to 4.89 and the calculated average value of this ratio was 3.80. All of them are clearly lower than the above-mentioned value of the N/S ratio recognized as optimal. Some researchers do not share the view that the N/S ratio is a reliable diagnostic tool for evaluating the S supply status. Their statement is based on the conviction that the surplus of one nutrient may be faultily interpreted as deficiency of another one, since a similar value of the ratio between two elements may be obtained at their completely different concentrations in the biomass. The results obtained in the presented studies show that high S supplementation significantly increases the wheat root N/S ratio due to a greater increase in the N than S content. In turn, the lower shoot N/S ratio was a consequence of the greater increase in the S than N content, whereas the changes in the N content under the highest S dose (9 mM) used in the experiment were insignificant. It is well documented that the N content in plant biomass increases with additional application of both N and S within a narrow range of the N/S ratio for optimum crop yield and quality (Jamal et al. 2010; Choong and Choong 2013). Our studies revealed that such a tendency was also true for the Ni-treated wheat intensive fertilized with S.

### Dry biomass and nickel accumulation

Our studies revealed that under the standard S dose (2 mM), Ni accumulation in the spring wheat cv. Zebra biomass increased together with the increasing concentration of this element in the nutrient medium. We also showed that the increase in Ni accumulation was much more pronounced in shoots than in roots, which led to more severe inhibition of growth and a decrease of shoot biomass than roots. Likewise in our study, a similar pattern of Ni distribution and accumulation in above- and underground parts of wheat as well as reduction of their growth was found by Kassim et al. (2013). Opposite trends were shown by Gajewska and Skłodowska (2008), Wang et al. (2009), Nafees and Amin (2014), and Stanišić Stojić et al. (2014). It should be stressed that by analyzing above- and underground parts of wheat, the contents of Ni (data in press) in Ni-stressed plants were much higher than the permissible limit of 2 mg kg^−1^ (Nafees and Amin 2014). It is well documented that growth inhibition and reduced dry biomass in the presence of Ni in the nutrient solution was a consequence of many processes, inter alia, changes in polysaccharides synthesis and carbohydrates translocation from shoots to roots (Kopittke et al. 2007); damage to the Golgi apparatus; changes in the ultrastructure of chloroplasts: disturbances in redox homeostasis; damage to DNA and RNA, proteins, and lipids; impaired cell divisions; suppression of cell elongation, e.g., via peroxidase activity stimulation; as well as disturbances in meristematic tissue differentiation and reduced intracellular species (Demchenko et al. 2005, 2010; Maksimović et al. 2007; Hansh and Mendel 2009; Jain et al. 2009; Kozhevnikova et al. 2009; Mesjasz-Przybyłowicz et al. 2010; Kumar et al. 2012; Parmar et al. 2012; Gopal 2014; Li et al. 2015). Also, our results (data in press) indicated increased superoxide anion radical (O_2_^−^) and hydrogen peroxide (H_2_O_2_) accumulation together with increased lipid peroxidation as the causes of suppressed root elongation.

Our studies revealed that Ni accumulation in wheat was determined by the concentration of this element in the nutrient solution as well as the S level and varied for the particular organs. Increased bioaccumulation of Ni in roots and shoots of wheat grown in the Ni-contaminated nutrient medium supplied with the 6 mM S level resulted from the greater increase in the biomass rather than the decrease in Ni in the dry weight (data in press). The increase in root Ni accumulation in the treatment 0.04 mM Ni/9 mM S was a consequence of the decreased Ni content and slight changes in the dry biomass. In turn, reduced Ni bioaccumulation in shoots of wheat stressed with high Ni doses and fertilized with 9 mM S resulted, to a great extent, from the decrease in the Ni content than the changes in dry biomass. In spite of the drop in the Ni content in the root and shoot biomass of plants treated with high Ni doses grown at 6 mM S as well as in roots at the treatment with 0.08 mM Ni/9 mM S, the Ni content in the biomass still exceeded the acceptable limits mentioned by Jabeen et al. (2010) (FAO/WHO standards), Shah et al. (2013), Nafees and Amin (2014), Nazir et al. (2015). However, the decrease in the Ni content corresponded with the increase in the organ biomass.

## Conclusion

In conclusion, it can be claimed that Ni presence in the nutrient medium reduces the biomass and disturbs the balance and accumulation of macronutrients in spring wheat cv. Zebra. Intensive S nutrition, especially with 6 mM S, at least partially increases the biomass, improves ionic equilibrium, and enhances nutrient accumulation in Ni-exposed plants in spite of increased Ni accumulation. Such an evident beneficial effect was not shown for the dose 9 mM S. Admittedly, the dose 9 mM S reduced Ni accumulation in shoots but increased accumulation thereof in roots. Compared to 6 mM, the dose 9 mM is less effective in improving the mineral status of the Ni-treated wheat. Both experimental intensive S doses (6 and 9 mM) resulted in an increased N and S content in root and shoots. A simultaneous drop in the K content in roots was shown and a rise in the Ca root content was recorded. Moreover, the dose 6 mM increased the shoot P content, but Mg 9 mM decreased the root Mg content. Given the promising results of the present study, further investigations concerning the influence of intensive S nutrition on metal-stressed plants are recommended.
